# Impact of Socio-Demographics and Knowledge, Attitudes, and Practices (KAP) on Misconceptions of Metformin Use in Diabetes: A Potential Myth and Disbelief in South Asia

**DOI:** 10.7759/cureus.67509

**Published:** 2024-08-22

**Authors:** Liyana Arachchi Chanika Rangani, Balapuwaduge Isuru Layan M Mendis, Harshini Rajapakse, Arosha Dissanayake

**Affiliations:** 1 Department of Nursing, University of Ruhuna, Galle, LKA; 2 Department of Medicine, University of Ruhuna, Galle, LKA; 3 Department of Psychiatry, University of Ruhuna, Galle, LKA

**Keywords:** metformin, compliance, adherence, medications, diabetes mellitus

## Abstract

Background: The influence of misconceptions and related socio-demographics on metformin use could hamper adherence to medications. This study aimed to assess the rates and causes of metformin non-adherence and to investigate knowledge, attitudes, and practices (KAP) on misconceptions of metformin use including the association with socio-demographic variables.

Methods: An observational analytical cross-sectional study was conducted at the diabetes clinic of Karapitiya Teaching Hospital in Galle, Sri Lanka. Causes of metformin non-adherence, associations with socio-demographics, and KAP on misconceptions on metformin use were assessed using the chi-squared test, t-tests, and ANOVA using IBM SPSS Statistics for Windows, Version 26.0 (Released 2019; IBM Corp., Armonk, New York, United States) (p<0.05).

Results: Metformin non-adherence was reported as 55%. Use of complementary and alternative therapies was 14.7%. Fear of major organ failure was the commonest (20.5%) reason quoted within the non-adherence group (N=223). Socio-demographic factors like ethnicity, lower education, unemployment, use of complementary and alternative therapies, and obtaining medications for other diabetes-related diseases significantly influenced adherence to the metformin-prescribed doses (p<0.05). Among all participants (N=400), the most common misconception was that long-term use of metformin caused organ damage (kidney 72.5%, liver 64.3%, and heart 34.8%), while 44% believed higher doses (two tablets or more for a day) caused organ damage. The KAP scores were reported as 24.5% with low, 52.7% moderate, and 22.7% satisfactory levels. Significantly lower KAP scores were associated with lower education levels and patients obtaining complementary and alternative therapies (p<0.05).

Conclusion: Misconceptions are not merely kept in mind but lead to non-adherence with metformin doses prescribed and warrant evidence-based educational interventions with the high-risk groups.

## Introduction

Sri Lanka is located in the South Asian region which has recorded higher prevalence rates of diabetes compared to other South Asian nations [1]. For instance, the prevalence of diabetes in South Asian countries was reported as follows: India, 11.4%; Bangladesh, 10.1%; Bhutan, 8.2%; the Maldives, 6.7%; Nepal, 9.2%; and Pakistan, 14.6% [2-5]. In Sri Lanka, there is an estimated 14.1 million adult population, 1.2 million of whom are projected to have diabetes. According to reports, the crude prevalence of diabetes reached a record high of 23% in 2019 [6]. Currently, diabetes is identified as a national priority by the Ministry of Health in Sri Lanka. Studies have found that the incidence of type 2 diabetes was reported higher among individuals from middle-class and lower-class backgrounds in urban, semi-urban, and rural locations [7]. Importantly, it has been reported that effective type 2 diabetes prevention and control among South Asians is hampered by attitudes, cultural differences, and social and religious beliefs [7].

Among the diabetes medications, metformin (1,1-dimethylbiguanide hydrochloride) remains the most widely prescribed diabetes medicine in the world [8]. In 1957, the French physician Jean Sterne first reported the successful use of metformin to treat diabetes [9-11]. In 1998, compelling research evidence was reported in the United Kingdom Prospective Diabetes Study (UKPDS) where metformin treatment caused reductions in any diabetes-related endpoint (32%), diabetes-related death (42%), and all-cause mortality (36%). Compared to other medications (i.e., insulin, thiazolidinediones, SGLT2 inhibitors, and sulfonylurea), metformin demonstrated significantly improved outcomes in any diabetes-related endpoint, all-cause mortality, and stroke. Moreover, metformin is available at a very low cost, making it highly cost-effective, especially considering the significant economic burden of diabetes complications. This financial benefit is especially crucial in resource-limited healthcare systems, where the affordability of metformin can play a key role in providing effective treatment to an increasing number of individuals in South Asian countries [12]. Later, the American Diabetes Association (ADA), International Diabetes Federation, and World Health Organization (WHO) endorsed and recommended the use of metformin as the first choice of medicine for all patients with type 2 diabetes [13-15].

Metformin lowers blood glucose levels by reducing intestinal glucose absorption, enhancing glucose uptake in tissues, inhibiting liver glucose production, and improving insulin sensitivity [16]. Metformin inhibits gluconeogenesis by blocking mitochondrial complex-I and activating AMP-activated protein kinase (AMPK), which plays a key role in energy and glucose metabolism [16]. Recent research has generated substantial evidence on the potential benefits and effects of metformin across various conditions, including polycystic ovarian syndrome, non-alcoholic fatty liver disease, cancer, aging, and neuroprotection, and its impact on the gut microbiome and transcriptome, particularly with miRNAs [17]. Nonetheless, metformin is associated with a low incidence of side effects. For example, the most common side effect reported so far is gastrointestinal symptoms, which include nausea, diarrhea, and vomiting (20-30%) [18,19]. A rare but known serious side effect is lactic acidosis (occurrence rate 1/30,000), which primarily affects diabetic patients who have liver and kidney dysfunction [20].

Though the scientific community has convinced itself on the efficacy and safety of metformin with robust scientific evidence, misconceptions about unwarranted side effects among patients in the community still persist [21]. Personal opinions, beliefs, and perceptions have strong influences on the lives of individuals and their ways of living including seeking treatment during illnesses [22]. The importance of understanding the misconceptions about chronic diseases such as diabetes, when providing excellent care and health education to both patients and healthy individuals, cannot be overstated. For instance, a published study reported that 20% of patients expressed a fear of developing drug-related side effects like renal failure with diabetes medications in Anuradhapura district, Sri Lanka [23]. However, the studies published were not able to explore the magnitude of this problem and evaluate its sociocultural determinants towards the management of the disease. Importantly, current misconceptions specifically related to metformin use except for the belief that it causes renal disease have not been studied in Sri Lanka. Therefore, the level of education, knowledge, and awareness of diabetes and its fear of medications require comprehensive investigations in national healthcare systems to prompt a strategic response in ensuring medication adherence and improved clinical outcomes of the disease.

Hence, the current study investigated the common misconceptions about metformin and its impact in terms of poor adherence to medications prescribed for diabetes. In pursuit of that, we aimed to assess the rates of non-adherence to prescribed medicines, causes of non-adherence, and knowledge, attitudes, and practices (KAP) on metformin-related misconceptions, along with the socioeconomic determinants. The findings of our study could help treating doctors identify patients with a higher likelihood of poor adherence and where greater emphasis on patient education and specific attention to dispelling misconceptions should be placed.

## Materials and methods

Study design and setting

**Figure 1:**
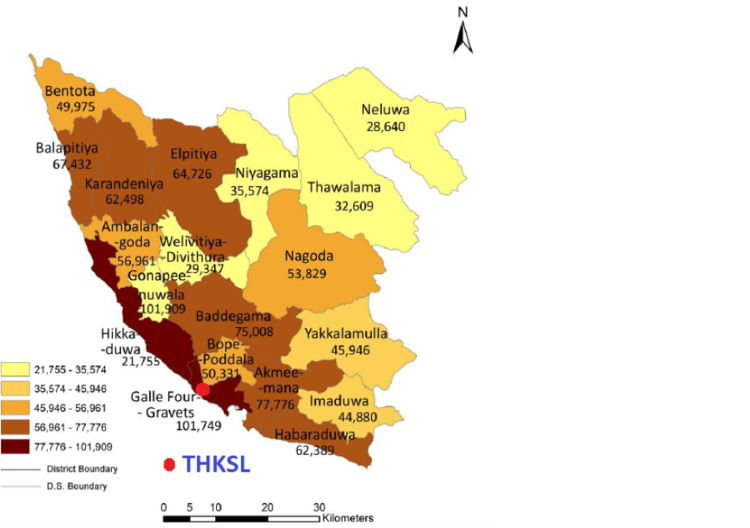
Galle Divisional Secretariat Division's population distribution

This was an observational analytical cross-sectional study conducted at the diabetes clinic of Karapitiya Teaching Hospital in Galle, Sri Lanka (THKSL). The THKSL is the largest tertiary care facility in the Southern Province and the third largest in the country [24]. The Southern Province accommodates a total population of 2,477,285 which is 12% of the country's population. In the Southern Province, the Galle district is the highest populated district comprising a population of 1,063,334 representing 42.9% of the Southern Province population (see Figure 1). A vast majority residing in the province receive healthcare facilities from THKSL [25].

Participants and sampling

The sample population constituted previously diagnosed patients with diabetes (fasting plasma glucose ≥126 mg/dL (7.0 mmol/L); two-hour plasma glucose ≥200 mg/dL (11.1 mmol/L) after an oral glucose tolerance test; or with a glycosylated hemoglobin A1c (HbA1c) ≥6.5% (48 mmol/mol)) which conformed with the diagnostic criteria stated by the ADA [26]. The formula used to calculate the sample size was as follows: N=p(1-p)Z^2^/d^2^. The sample size was calculated as 384 using a 1.96 standard deviation for a 5% alpha error. As the prevalence of multiple characteristics related to diabetes that were to be assessed is not known, the sample size calculation was conducted using a larger sample. The maximum sample size is obtained if the anticipated prevalence was estimated as 50% as per the WHO publication by Lwanga and Lemeshow in 1991. Hence, the anticipated population proportion was taken as 50% in order to achieve the maximum sample size. Adding a 5% dropout rate, the final sample was calculated to be 404 subjects [27]. A consecutive sampling method was used to recruit patients who sought treatment from the diabetes clinic in both morning and afternoon clinics at the THKSL.

Inclusion criteria and exclusion criteria

Patients who were taking metformin and were aged 20 years or more were recruited. When recruiting patients, recent clinic books and records were checked to verify the patients who met the ADA diagnostic criteria with diabetes. Patients less than 20 years old were excluded as type 2 diabetes mellitus (T2DM) is a rare disease in those under 20 years. Also, the majority of less than 20-year-old diabetic subjects are diagnosed with type 1 diabetes mellitus (T1DM), and metformin is not routinely used in T1DM. Additionally, medication adherence of those under 20 years is influenced by many multitudes of psychological, social, and cultural factors. As these themes are not explored in this paper, the age cutoff was taken beyond the teen years from 20 onwards. Non-consented subjects were excluded from the study. Moreover, patients suffering from end-stage kidney and liver failures were also excluded including patients with severe psychiatric disorders who were unable to answer our questionnaire.

Data collection

Trained medical officers and research assistants recruited the patients. Informed written consent was obtained before data collection from each participant. Clarifications and problems raised by the subjects during the interview were addressed at the clinic.

Study instruments

An interviewer-administered questionnaire was used to collect data. The questionnaire comprised three parts: the first part (Appendix A) focused on demographic data, the second part (Appendix B) contained the medical history of the patient, and the third and final part of the questionnaire (Appendix C) comprised a seven-item mini-questionnaire that assessed the KAP on misconceptions about metformin use which would eventually become one's own opinions, beliefs, and perceptions. The literature review found no existing validated KAP scale for metformin use. Therefore, a new scale was created for this study, focusing on key factors. This scale, or a refined version, could be used by future researchers for similar studies. The researchers developed a questionnaire exploring metformin-related myths/misconceptions and long-term use (regular use of metformin for more than six months), some of which had been identified as previously published literature on metformin use [21,28-31]. The patients were interviewed in the vernacular. The linguistic validation process was conducted using standard and systematic procedures, following cross-cultural translation guidelines, to translate the English KAP questionnaire into Sinhalese. Two professional Sinhalese translators carried out the forward translation. A bilingual psychiatrist and a consultant physician, both unfamiliar with the scale, reviewed the Sinhalese version for appropriateness. The reconciled translation was then compared with the original English questionnaire for similarity. Finally, two independent translators, who had not seen the original, performed a back translation, and any disparities were addressed to ensure consistency with the original scale. Table 1 demonstrates the English version of the questionnaire (Part 3).

**Table 1:** Questionnaire on KAP of metformin use KAP: knowledge, attitudes, and practices

Questions	Yes	No	Don't know
(1) Metformin has a lot of side effects	-1	+1	0
(2) Metformin causes blood sugar levels to reduce to lower-than-normal levels	-1	+1	0
(3) Metformin dose can be reduced after eating bitter gourd (karavila) or crepe ginger plant (thebu kola or *Costus speciosus*) or else other herbs	-1	+1	0
(4) Long-term use of metformin causes kidney failure	-1	+1	0
(5) Long-term use of metformin causes liver failure	-1	+1	0
(6) Long-term use of metformin causes heart failure	-1	+1	0
(7) High doses of metformin (more than two tablets) cause kidney and liver failure	-1	+1	0
KAP score total	-7	+7	0

**Figure 2:**
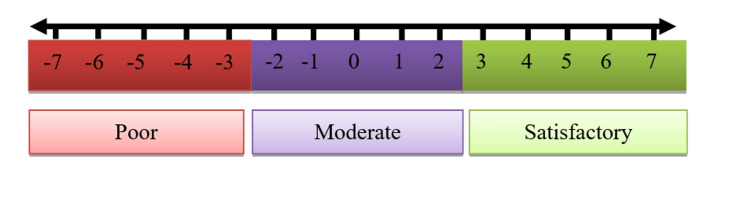
Numeric scale of assessing KAP of metformin use KAP: knowledge, attitudes, and practices

For each question, three answers were provided, namely, "Yes," "No," and "Don't know." Patients' KAP score was calculated based on answers to the questions relating to misconceptions of metformin. One point (+1) was offered for each correct response, one point was reduced to wrong answers (-1), and no points (0) were given to "Don't know" answers. The total points of each question were summated and assigned a final score. Scores ranging from -7 to -3 were considered with a "Poor" KAP level, -2 to +2 with a "Moderate" KAP level, and +3 to +7 with a "Satisfactory" KAP level (see Figure 2). The final scores of the numeric scale were assigned into three numeric intervals representing the respective knowledge categories. Therefore, the scale provided an equal probability for any score of knowledge to be selected.

Data analysis

Descriptive data were presented with frequencies, percentages, and means. Associations of socio-demographic variables were performed using chi-squared tests using 2×2 and 3×2 tables at 0.05 confidence level. Independent t-test and ANOVA were used to find the associations with socio-demographic variables. All the data was analyzed using IBM SPSS Statistics for Windows, Version 26.0 (Released 2019; IBM Corp., Armonk, New York, United States).

## Results

Descriptive analysis of socio-demographic data and medical history

A total of 400 consecutive diabetes clinic attendees with metformin treatment and prescription were interviewed. The majority were females with 78.5% and belonged to the 41-60-year age category. Considering educational level, 51.7% of subjects were educated up to General Certificate of Education-Ordinary Level representing grades 1-11 (6-16 years old). Importantly, a substantial number of patients (61.5%) were unemployed while 52% with a family history of diabetes. Patients with diabetes for more than 10 years accounted for 22.2% of patients, while 63% were diagnosed for a period between one and 10 years. In addition to the metformin treatment, 37.75% of patients received other diabetes-related medications, insulin (18.5%) and other oral hypoglycemic agents (19.2%), while the majority (62.2%) with metformin prescription only. A total of 59 patients (14.75%) were receiving additional complementary and alternative therapies, like herbal remedies and Ayurveda treatments. A sizeable proportion of the sample receives additional medications for other diabetes-related complications: cardiovascular disease (CVD) 47% and chronic liver and kidney diseases 2.7% and 7.5% with other complications.

Descriptive analysis of the causes of metformin non-adherence

**Figure 3:**
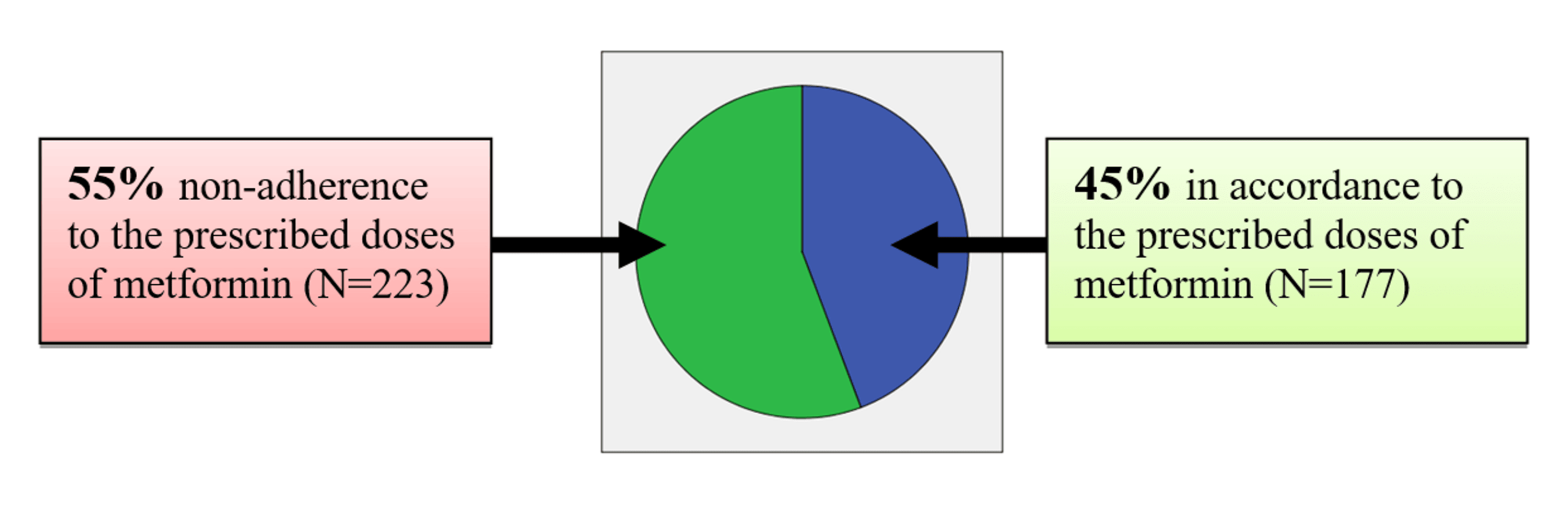
Adherence to the prescribed doses of metformin

Out of 400 patients, a significant number (55%) reported not taking metformin as directed (see Figure 3). Among the non-adherent patients to metformin-prescribed doses (N=223), perceived fear of developing a major organ failure such as kidney, liver, and heart failures was reported as commonly quoted misconceptions of metformin at 20.5%. The second most commonly quoted reason was "forgetting medications" with a percentage of 8.5%. Perceptions of side effects (i.e., feeling weak, abdominal discomfort, nausea, and vomiting) were some of the reasons stated only by 6.8%. Importantly, 6.5% of patients reduced their medication dose based on self-glucose monitoring when they believed their blood sugar levels were normal. Table 2 indicates the frequencies and percentages of reasons quoted for metformin non-adherence to the prescribed doses.

**Table 2:** Causes for metformin non-adherence to the prescribed doses

Reason quoted (N=223; 55%)	Frequency (n)	Percentage (%)
Fear of major organ failure such as kidney, liver, or heart failure	82	20.5
Forgetting to take the medication	34	8.5
Adopting home remedies and taking special herbals perceived as lowering blood glucose	31	7.8
Side effects of metformin ("feeling weak, abdominal discomfort, nausea, and vomiting")	27	6.8
Self-blood glucose monitoring and lowering dose when having satisfactory glucose values	26	6.5
Taking additional allopathic glucose-lowering medicines	23	5.8

Associations of metformin use with the socio-demographic variables

There was no significant association reported with gender, age groups, family history, duration of illness, and consuming other diabetes drugs on metformin compliance to the recommended doses (p<0.05). However, significant associations were obtained for ethnicity, educational levels, employment status, obtaining complementary and alternative therapies, and taking medications for diabetes-related other complications and diseases. Table 3 represents the metformin use and respective socio-demographic associations dispersed across the sample.

**Table 3:** Baseline characteristics of the patients and associations with metformin use Note: p-values with * mark indicate significant associations

Socio-demographic characteristics of patients	Category	Frequency (n)	Percentage (%)	Use of metformin according to the recommended dose		
				Yes (%)	No (%)	Significance (p<0.05)
Gender	Female	314	78.5	44.6	55.4	0.79
	Male	86	21.5	43	57	
Age groups (in years)	20-40	37	9.25	56.8	43.2	0.13
	41-60	185	46.25	45.9	54.1	
	61-80	178	44.5	39.9	60.1	
Ethnicity	Sinhala	323	80.75	41.2	58.8	0.01*
	Muslim	77	19.25	57.1	42.9	
Education levels	Less than ordinary level	207	51.7	33.3	66.7	0.00*
	Up to ordinary level	122	30.5	52.5	47.5	
	Up to advanced level	66	16.5	59.1	40.9	
	Diploma or higher	5	1.3	100	-	
Employment status	Employed	154	38.5	50.6	49.4	0.04*
	Unemployed	246	61.5	40.2	59.8	
Family history of diabetes	Positive	208	52	43.3	56.7	0.68
	Negative	192	48	45.3	54.7	
Duration of illness	<1	59	14.75	49.2	50.8	0.67
	1-10	252	63	42.9	57.1	
	>10	89	22.25	44.9	55.1	
Use metformin medication and other diabetes medications	Yes (with other drugs)	151	37.75	41.1	58.9	0.31
	No (metformin only)	249	62.25	46.2	53.8	
Taking additional complementary and alternative medications (i.e., herbal remedies and Ayurveda medication for diabetes)	Yes	59	14.75	28.8	71.2	0.01
	No	341	85.25	46.9	53.1	
Taking medications for diabetes-related other complications and diseases	Yes	240	60	40	60	0.03
	No	160	40	50.6	49.4	

Assessment of KAP on metformin misconceptions

Descriptive frequencies reported for the metformin KAP questionnaire are presented in Table 4. Results revealed that 33.3% possessed a belief that metformin is a medication with many side effects, while 31.8% had the misconception that metformin results in hypoglycemia. It was also believed that long-term metformin use (more than six months) caused kidney failure and liver failure by 72.5% and 64.3%, respectively. A further 44.3% believed metformin taken at high doses can cause kidney failure. Importantly, 18% held a perception that metformin doses can be reduced on the days they consume bitter gourd (karavila) or *Costus speciosus* (thebu kola) or else herbal foods.

**Table 4:** Descriptive frequency percentages of the KAP questionnaire KAP: knowledge, attitudes, and practices

Questions	Yes (%)	No (%)	Don't know (%)
(1) Metformin has many side effects	33.3	59.7	7.0
(2) Metformin causes the blood sugar level to reduce more than the normal blood sugar level	31.7	65.3	3.0
(3) Metformin dose can be reduced after eating bitter gourd (karavila) or *Costus speciosus* (thebu kola) or else herbal foods	18	79.7	2.3
(4) Long-term use of metformin causes kidney failure	72.5	22.3	5.2
(5) Long-term use of metformin causes liver failure	64.3	25.3	10.4
(6) Long-term use of metformin causes heart failure	34.8	27.8	37.4
(7) High doses of metformin (more than two tablets) cause kidney and liver failure	44.3	34.3	21.4

Assessment of KAP scores and its associations with the socio-demographic variables

**Figure 4:**
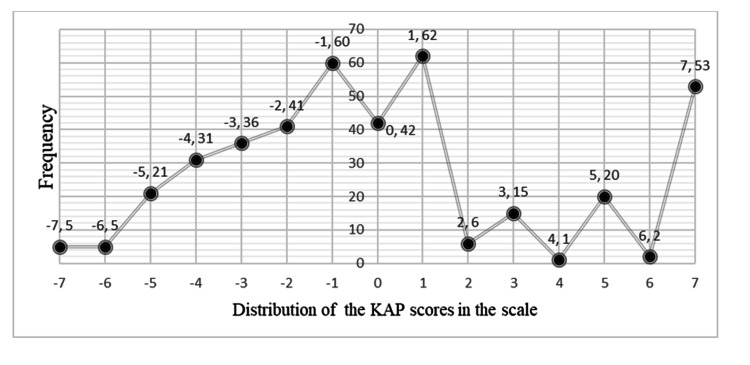
Frequency distribution of KAP scores KAP: knowledge, attitudes, and practices

Using the seven-item questionnaire, KAP scores were calculated and assigned for each subject. The distribution of the KAP scores for each score from -7 to +7 is presented in Figure 4. Although KAP scores of 60% of patients were equal or below 0, a considerable proportion of subjects (13.25%) provided the correct answers for all questions scoring +7. Considering knowledge categories, there were 24.5% of patients with low KAP scores (-7 to -3: poor KAP level), 52.7% with moderate KAP scores (-2 to +2: moderate KAP level), and 22.7% with satisfactory KAP scores (2 to 7: satisfactory KAP level). The mean KAP score of the sample population was reported as +0.15 which represents a moderate KAP level.

Table 5 presents the associations of KAP scores with the patient characteristics. Findings revealed that there was no significant difference in the mean KAP scores of males (0.45±3.71) and females (0.07±3.66) (p<0.05). Despite the lower KAP scores in the 61-80-year-old age group, the KAP scores were not significantly different across the three age categories. Similarly, ethnicity had not significantly impacted KAP scores between the Sinhalese and Muslims. Essentially, the level of education reported significant associations with the KAP scores. The patients with higher education reported a profound KAP score of 5.6±2.60. Employment status, family history, and duration of illness obtained non-significant associations with the KAP scores. Obtaining concurrent complementary and alternative therapies (i.e., Ayurveda and herbal treatments) was another factor that was significantly associated with a lower KAP score (p<0.00). Patients receiving treatments for diabetes-related other diseases and complications reported non-significant associations with the KAP scores.

**Table 5:** Associations of demographic variables of patients with the KAP scores Note: p-values with * mark indicate significant associations KAP: knowledge, attitudes, and practices; SD: standard deviation

Demographic characteristics of patients	Groups	Mean KAP score	SD (±)	P-value
Gender	Female	0.07	3.66	0.39
	Male	0.45	3.71	
Age (in years)	20-40	0.67	3.39	0.42
	41-60	0.28	3.74	
	61-80	-0.08	3.66	
Ethnicity	Sinhala	0.00	3.59	0.10
	Muslims	0.81	3.93	
Education levels	Less than ordinary level	-4.58	3.46	0.00*
	Up to ordinary level	0.55	3.63	
	Up to advanced level	0.92	3.93	
	Diploma/higher or above	5.6	2.6	
Employment status	Employed	0.45	3.73	0.2
	Unemployed	-0.03	3.63	
Family history	Positive	0.00	3.49	0.38
	Negative	0.32	3.67	
Duration of illness	Less than 10 years	0.49	4.14	0.58
	1-10 years	0.01	3.59	
	More than 10 years	0.33	3.59	
Complementary and alternative medications (i.e., herbals/Ayurveda treatments)	Yes	-1.28	3.46	0.00*
	No	0.4	3.65	
Medications for diabetes-related other complications and diseases	Yes	0.07	±3.69	0.59
	No	0.27	±3.65	

## Discussion

The current study is the first attempt to investigate the impact of socio-demographical factors, causes, and KAP leading to misconceptions about metformin use in Sri Lanka. Findings revealed that 55% of the patients were non-adherent to the metformin-prescribed doses. Similarly, a recent study conducted in the Anuradhapura district reported poor overall medication adherence among 60.3% of diabetic subjects [23]. Subsequently, the study revealed that "troublesome symptoms and not feeling like continuing treatment" (22.1%), "fear of developing drug-related side effects like renal failure" (21.3%), and "too complex medication regimens" (14.9%) were some of the causes associated with poor adherence [23]. Essentially, the current study revealed that the co-existence of poor health literacy and a wide array of misconceptions has led to metformin non-adherence. Though a practicing physician/diabetologist is likely to believe that the commonest reason for poor adherence to metformin would be its troublesome gastrointestinal side effects, this research study demonstrates that this was the case only in 6.8% of those who were non-adherent (see Table 2).

For example, fear of major organ failures (20.5%), forgetting medications (8.5%), taking home remedies (7.8%), perceptions of metformin-related side effects (6.8%), and tailoring medications according to the glucose levels (6.5%) were identified as most commonly quoted causes among non-adherent patients (N=223). There has been no systematic study on the frequency of concomitant alternative and complementary therapy in addition to clinic-prescribed medications for diabetes. Of the 400 patients studied, 14% use alternative and complementary treatments alongside the clinic-prescribed medications. This opens up a vast scope for study on the combined effects of these therapies, pharmacological interactions, and potential enhancement of target organ damage in the future.

Myths and misconceptions abound in diseases like diabetes because of the chronicity of the illness, severe comorbidities, and complications as well as the expectations on allopathic Western medicine practitioners to provide a permanent cure that most patients seek [21,22]. In this study, the most commonly recorded misconception on metformin use was its "long-term use" causing kidney failure (72.5%) and liver failure (64.3%) and misconceptions on "higher doses" (more than two tablets) resulting in liver and kidney failure (44.3%). The misconceptions in our study were consistent with a previously published study in Sri Lanka which reported a significant proportion of patients believed that metformin causes renal damage (37.8%) and taking insulin was harmful (21.5%) [30]. This could be because metformin is discontinued when advanced renal failure is detected due to concerns of the rare side effect lactic acidosis [29]. Also, in the past, metformin has been treated with suspicion primarily due to its closely related drug phenformin which was banned for causing lactic acidosis [9]. However, metformin can be used safely in mild renal failure (GFR <30) according to the evidence-based guidelines [32].

Significant associations were recorded for metformin poor adherence with some ethnic groups, educational level, unemployment, obtaining complementary and alternative therapies, and taking medications for diabetes-related other complications and diseases. The results of our study were consistent with the studies conducted in India and Pakistan where the investigators identified poor educational level, unemployment, and spiritual belief were significantly associated factors [21,28].

The mean KAP score of the sample was reported as +0.15, and the results of KAP scores revealed only 22.7% with satisfactory KAP scores, while the majority belonged to the moderate KAP category. This indicates the existence of moderate to higher levels of metformin-related misconceptions among the sample population. The associations of KAP scores and socio-demographic variables were computed and revealed significant associations between education levels and use of complementary and alternative treatments. A study conducted in North India reported a higher existence of misconceptions among females and non-educated groups [28]. In contrast, our study demonstrated that there was no significant difference in the KAP scores of males and females. However, our study demonstrated higher KAP scores in the highly educated patient group eliciting the existence of lower misconceptions among the educated group.

Importantly, lower KAP scores were reported with patients who sought complementary and alternative treatments (Ayurveda and herbal treatments). This indicates patients seeking alternative ways and means of diabetes treatments could possess higher existence of misconceptions and lower faith in Western allopathic medicine [28]. Many believe that herbal medicines are effective in the treatment/cure of diabetes. Some believe that spiritual activities help cure diabetes. These sections of people often present late to doctors with severe hyperglycemic episodes and complications as they first seek spiritual or herbal treatment [21]. While certain herbal mixes had substantial benefits in decreasing blood sugar, scientists are unable to conclude with certainty about their effectiveness because of methodological flaws and limited sample sizes. Although no significant side effects have been observed, additional investigation needs to be conducted as there is currently little evidence to support the use of these therapies in standard clinical practice [33].

An exponential rise in myths and misconceptions could be expected in the digital age and unrestricted self-publishing. For example, a study conducted in Germany, Sweden, the Netherlands, the United Kingdom, and the United States revealed the misconceptions and myths even with insulin treatments [34]. As these misconceptions were generally held among all patients, even more than for individual patients, community-level education is needed to dispel these widely prevalent myths. As health literacy was lower in those with lower education, rather than the traditional educational methods of producing leaflets and booklets, digital media could be used effectively to convey simple messages. 

The study highlights the magnitude of the problem clinicians treating patients with diabetes with metformin are facing. Poor adherence is high, and that needs to be enquired at clinical encounters and concerns addressed. Myths are prevalent, and impactful, simple methods that reach out even to those with lower educational attainments need to be developed. Future research needs to answer two questions. The first is: "How do patients acquire these misconceptions?". When a patient has been on metformin for diabetes and subsequently develops chronic kidney disease (CKD), doctors discontinue metformin. Perhaps due to poor communication, perhaps due to misinformation, the patient may link up the two and start to believe that long-term use of metformin caused the CKD and that is why the doctor stopped it. Is this the basis of the renal failure myth? The second question future research needs to answer is: "What are the most useful health literacy-increasing educational interventions that can address these problems?". The findings of this study lay a foundation to build further research upon.

Limitations

The methodology adopted had inherent deficiencies. The proportion of females attending the clinic was substantially higher (78%). Most males are employed during the daytime when these clinics are held. Whether having a higher proportion of females being interviewed had an impact on the findings is difficult to ascertain. But it is very likely that what we have revealed are highly abundant misconceptions shared at the family and society level. Obstacles to obtaining accurate information arose because some patients worried about discussing aspects of poor adherence. They felt that revealing this would upset treating doctors. Anyhow, the patients were assured that the information they provided was treated with the utmost confidentiality and would not be revealed to their doctors or any other party and thus their future treatment plans would not be affected. The levels of non-adherence and prevalence of misconceptions detected by the study are very high, and this methodological limitation raises the possibility that these staggeringly high figures themselves could be an understatement and the actual figures may be even higher.

The study posed a particularly challenging ethical dilemma to the researchers. The dilemma was not revealing to the treating doctors that some of their efforts were in vain as the patients admitted to the researchers that they were taking metformin in doses less than prescribed. The other ethical dilemma was knowing that the patients were coming to harm by non-adherence and not correcting it. Ethical duties of patient confidentiality, beneficence, and non-maleficence were present and in contradiction. As maintaining patient confidentiality was the primary duty, the researchers offered another opportunity to participants to discuss any worries they had about metformin use with a member of the medical team. Further, a leaflet was sent to participants at the end of the research setting out the true factual situation regarding use of metformin. 

## Conclusions

The majority of patients who are prescribed metformin for diabetes are non-compliant with the doses prescribed and this is a result of a multitude of myths and misconceptions surrounding its use and the fear of major organ failure is the foremost among them. Health literacy related to metformin use is poor among those with diabetes and needs focused educational interventions from treating clinicians to correct them. Patient outcomes will be less than optimal unless dispelling metformin-related myths and misconceptions is recognized as a matter of urgency.

## Appendices

Appendix A

**Table 6:** Questionnaire on demographic data: Part A

Description	Answers	Response (√/×)
Gender	Male	
	Female	
Age group (in years)	20-40	
	41-60	
	61-70	
Education level	Less than ordinary level	
	Up to ordinary level	
	Up to Advanced level	
	Higher education and above	
Employment status	Employed	
	Unemployed	

Appendix B

**Table 7:** Questionnaire on medical history relating to diabetes mellitus: Part B

Description	Answers
State the family history of diabetes mellitus (parents/siblings) or no one with diabetes	
How long do you have diabetes mellitus?	
Do you take metformin?	
How many tablets do you take per day?	
Are you taking the recommended metformin dose or else are you taking tablets less than prescribed?	
If so, why?	
Are you taking other tablets for diabetes mellitus?	
Do you use home remedies or traditional treatment for diabetes mellitus?	
What are those remedies?	
Do you have kidney disease, heart disease, or stroke?	
Do you have any other worries?	

Appendix C

**Table 8:** Questionnaire on KAP of metformin use: Part C KAP: knowledge, attitudes, and practices

Description	Yes	No	Don't know
Metformin has a lot of side effects			
Metformin causes blood sugar levels to reduce to lower-than-normal levels			
Metformin dose can be reduced after eating bitter gourd (karavila or *Momordica charantia*) or crepe ginger plant (thebu kola or *Costus speciosus*) or else other herbs			
Long-term use of metformin causes kidney failure			
Long-term use of metformin causes liver failure			
Long-term use of metformin causes heart failure			
High doses of metformin (more than two tablets) cause kidney and liver failure			
